# Supplementary material for: Microporous Activated Carbon from Pisum sativum Pods Using Various Activation Methods and Tested for Adsorption of Acid Orange 7 Dye from Water
Source: Molecules. 2022 Jul 28;27(15):4840. doi: 10.3390/molecules27154840 (PMC9369958; doi:10.3390/molecules27154840)
Supplement: Supplementary file 1 [file molecules-27-04840-s001.zip › molecules-1819507-supplementary.pdf]

Microporous Activated Carbon from *Pisum sativum* Pods Using Various Activation Methods and Tested for Adsorption of Acid Orange 7 Dye from Water

Mohamed A. El-Nemr [1], Ahmed El Nemr [2,*], Mohamed A. Hassaan [2], Safaa Ragab [2], Luigi Tedone [3], Giuseppe De Mastro [3] and Antonio Pantaleo [3]

[1] Department of Chemical Engineering, Faculty of Engineering, Minia University, Minia, Egypt; mohamedelnemr1992@yahoo.com
[2] National Institute of Oceanography and Fisheries (NIOF), Marine Pollution Lab, Alexandria 21556, Egypt; mhss95@mail.com (M.A.H.); safaa_ragab65@yahoo.com (S.R.)
[3] Department of Agriculture and Environmental Sciences, Bari University, 70121 Bari, Italy; luigi.tedone@uniba.it (L.T.); giuseppe.demastro@uniba.it (G.D.M.); antonio.pantaleo@uniba.it (A.P.)
* Correspondence: ahmedmoustafaelnemr@yahoo.com or ahmed.m.elnemr@gmail.com

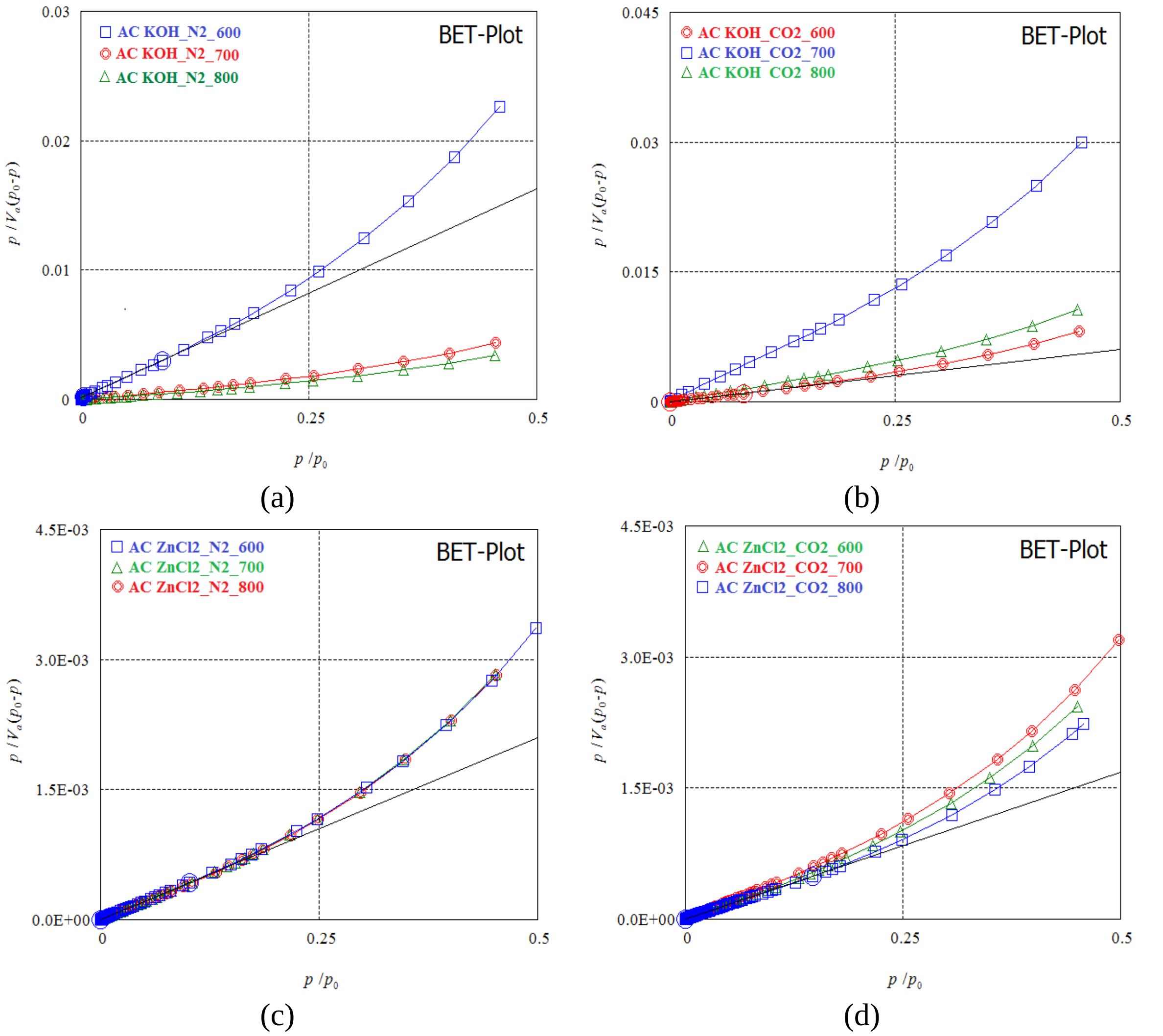

Figure S1. BET analysis of AC prepared from pea pods impregnated with using (a) KOH and carbonized under N_2 gas flow, (b) KOH and carbonized under CO_2 gas flow, (c) $ZnCl_2$ and carbonized under N_2 gas flow, and (d) $ZnCl_2$ and carbonized under CO_2 gas flow.

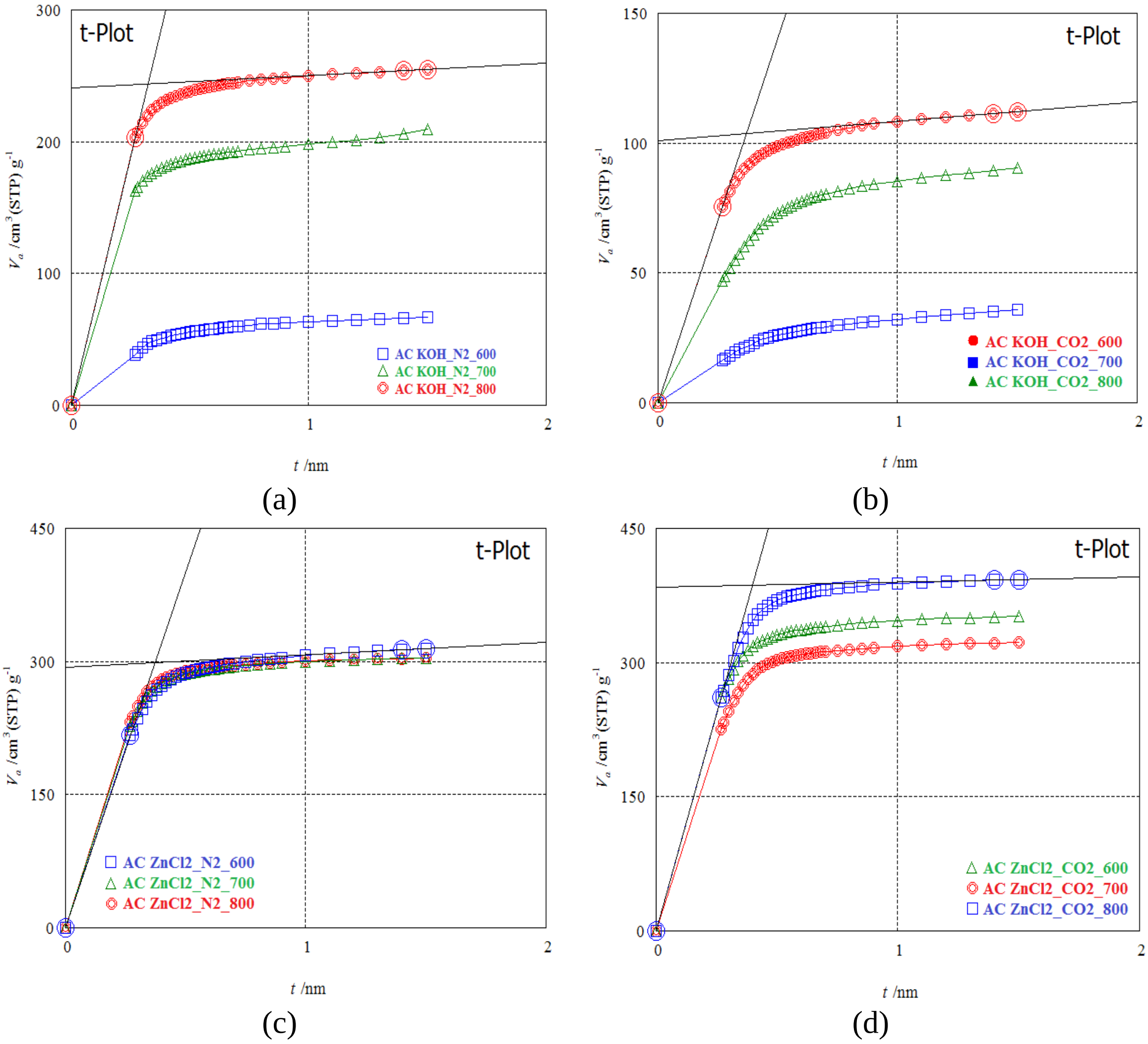

Figure S2. The t-plot analysis of AC prepared from pea pods impregnated with using (a) KOH and carbonized under N_2 gas flow, (b) KOH and carbonized under CO_2 gas flow, (c) $ZnCl_2$ and carbonized under N_2 gas flow, and (d) $ZnCl_2$ and carbonized under CO_2 gas flow.

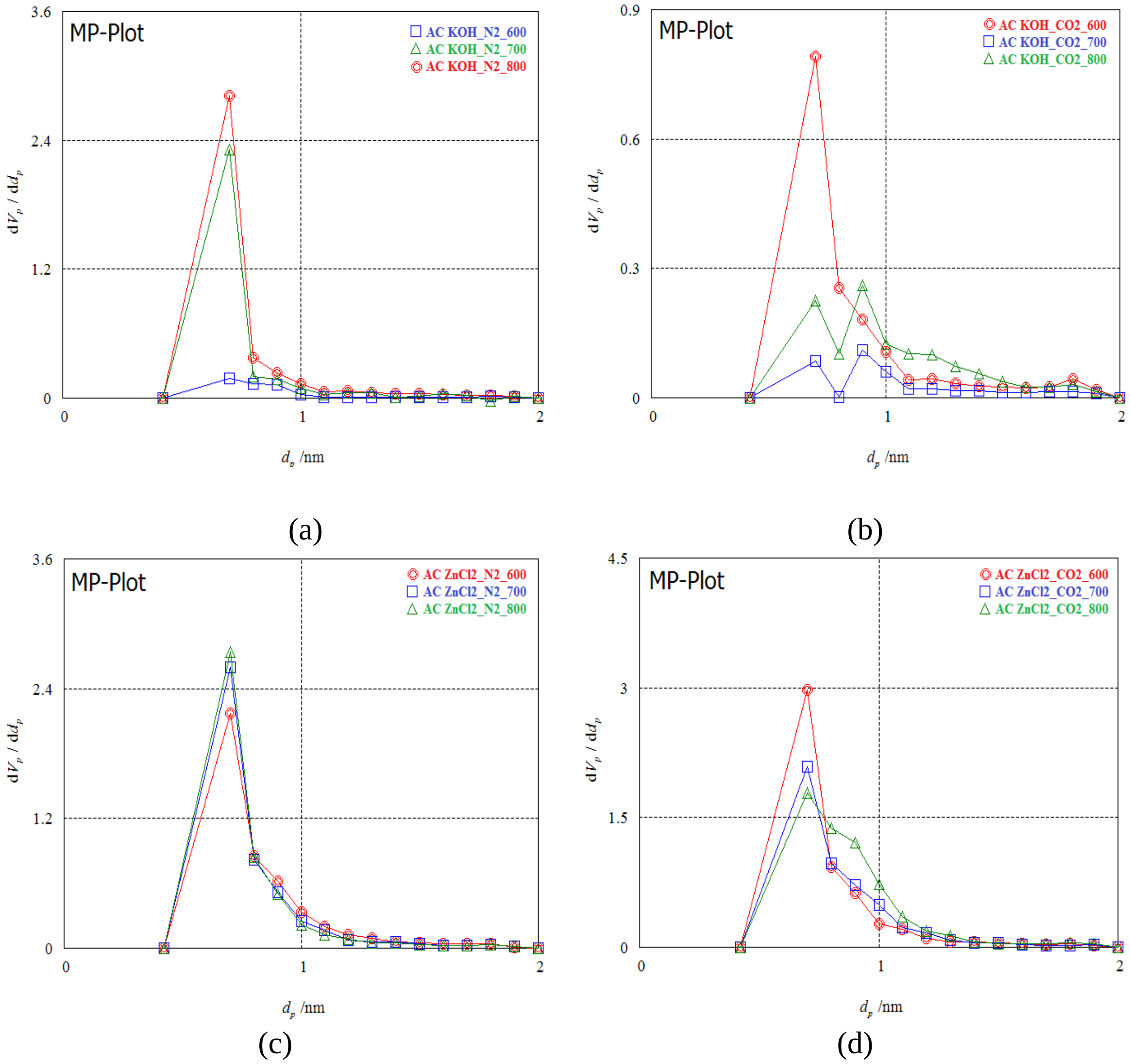

Figure S3. (a) MP analysis of AC KOH-N_2 (b) MP analysis of AC KOH-CO_2, (c) MP analysis of AC $ZnCl_2$-N_2 (d) MP analysis of AC $ZnCl_2$-CO_2.
